# Key metabolites associated with the onset of flowering of guar genotypes (*Cyamopsis tetragonoloba* (L.) Taub)

**DOI:** 10.1186/s12870-020-02498-x

**Published:** 2020-10-14

**Authors:** Serafima Arkhimandritova, Alexey Shavarda, Elena Potokina

**Affiliations:** 1 N.I. Vavilov Institute of Plant Genetic Resources (VIR), St. Petersburg, Russia; 2 Komarov Botanical Institute, St. Petersburg, Russia; 3 Saint Petersburg State University, St. Petersburg, Russia; 4 Saint Petersburg State Forest Technical University, St. Petersburg, Russia

**Keywords:** GC-MS-analysis, Metabolomics, Flowering time, Guar, *Cyamopsis tetragonoloba* (L.) Taub., Biomarkers

## Abstract

**Background:**

Guar (*Cyamopsis tetragonoloba* (L.) Taub.), a short-day plant, is an economically valuable legume crop. Seeds of guar serve as a source of galactomannan polysaccharide, known as guar gum, which is in demand in the gas and oil industries. The rapid and complete maturation of guar seeds depends on the flowering time of a particular genotype. It is known that flowering in guar is controlled by several gene systems. However, no information about the process and mechanisms that trigger flowering in guar on the molecular and biochemical levels was previously reported. The aim of the study was to investigate the metabolic landscape underlying transition to the flowering in guar using GC-MS-metabolomic analysis.

**Results:**

82 diverse guar genotypes (each in 8 replicates) from the VIR collection were grown under experimental conditions of high humidity and long photoperiod. In the stress environment some guar genotypes turned to flowering early (41 ± 1,8 days from the first true leaf appearance) while for others the serious delay of flowering (up to 95 ± 1,7 days) was observed. A total of 244 metabolites were detected by GC-MS analysis on the third true leaves stage of 82 guar genotypes. Among them some molecules were associated with the transition of the guar plants to flowering. Clear discrimination was observed in metabolomic profiles of two groups of «early flowering» and «delayed flowering» plants, with 65 metabolites having a significantly higher abundance in early flowering genotypes. Among them 7 key molecules were identified by S-plot, as potential biomarkers discriminating of «early flowering» and «delayed flowering» guar genotypes.

**Conclusions:**

The metabolomic landscape accompanying transition to flowering in guar was firstly described. The results obtained can be used in subsequent genomic research for identifying metabolite-gene associations and revealing genes responsible for the onset of flowering and photoperiod sensitivity of guar. In addition, the detected key metabolites associated with flowering of guar can be employed as biomarkers allowing rapid screening of breeding material for the potentially early flowering genotypes.

## Background

Guar is a short-day legume crop that recently became popular since its seeds serve as is a source of galactomannan polysaccharide (the guar gum), which is used in many industries including gas and oil production. Guar tolerates high temperatures and dry conditions and it is well adapted to arid and semi-arid climate of India and Pakistan [1]. Several attempts have been made to introduce the economically valuable legume crop to the countries of the higher geographical latitudes. The main problem was repeatedly reported when introducing guar to the new habitats - an excessive length of the crop cycle creating problems at harvest (e.g. [2]). For US the growing season of guar was reported from 60 to 90 days (determinate varieties) to 120–150 days (indeterminate varieties), and only the earliest-maturing guar varieties are recommended for production in Wisconsin and Minnesota [1]. Besides the determinate and indeterminate growth habits, a particular daylight length significantly affects the onset of flowering in guar [3]. In turn, day-neutral guar genotypes usually mature earlier than those with high sensitivity to the length of photoperiod [4].

Elucidation of the genetic control of the onset of flowering in guar can significantly benefit from the use of metabolite profiling as a new tool of functional genomics. There are several reports evidenced that each plant genotype possesses a distinct metabolic profile (e.g. [5–7]. The metabolomic profiling has the potential not only to provide deeper insight into complex regulatory processes, but also to determine phenotype directly [5].

Metabolic analysis coupled with genomic studies have been repeatedly carried out in many plant species. For example, using the metabolic approach, genetic factors related to pest resistance in carrots [8], tomatoes [9] and to salt stress of barley [10] were determined. Several studies have been devoted to the search for molecular mechanisms that significantly reduce the sensitivity of crops to high temperature, drought, salinization, high metal content, and some genes underlying resistance to the abiotic stressors have been revealed [11–14]. For guar, however, the metabolic approach was used so far only to study the antimicrobial activity of seeds [15, 16], seeds qualitative composition [17] and seeds medicinal properties [18]. Out of the “omics” approaches, transcriptome profiling of leaf tissues of two guar varieties has recently been reported, providing information on more than 62 thousand unigenes [19]. Employment of metabolomic profiling as an additional tool for functional genomics could provide a new understanding of metabolism of plants and its interaction with the environment [20, 21].

The aim of the study was to analyze the metabolic landscape underlying transition of the various *C. tetragonoloba* genotypes to flowering under long daylight conditions, which are stressful for this species of short-day plants. To achieve the task, we conducted a six-month vegetation experiment to grow 96 different guar genotypes in a greenhouse, with a natural daylight length corresponding to the geographical latitude of St. Petersburg (~ 60°N). We examined how different genotypes were segregating by their onset of flowering, depending on their individual sensitivity to the photoperiod. At the same time, for each plant, we performed metabolomic profiling of tissues of the third true leaf - the developmental stage that precedes the formation of the flowering bud.

## Results

### Variation of flowering time among the different guar genotypes at the long photoperiod under greenhouse conditions

Guar – is a short day plant, which means that flowering of guar is accelerated by daylight length shorter than the critical photoperiod [3]. The optimal length of the photoperiod during the growing season of guar varies from 12.7 h to 13.8 h, as in Jodhpur province (India), where this crop is widely cultivated. In our experiment 96 guar genotypes, each presented by 8 individuals, have been grown under conditions of the greenhouse of the Pushkin branch of VIR during six months (May – October) at the photoperiod that is natural to the latitude of St. Petersburg (59°53′39″N). The experiment allowed us to monitor the reaction of different genotypes of the short-day crop to a gradually decreasing length of daylight: from the maximum (~ 19 h) on the day of the summer solstice, to a relatively short (11 h) in the first decade of October [4]. We had an opportunity to observe how the guar plants one by one passed to flowering as soon as the photoperiod reached a certain threshold level specific for a particular genotype. This allowed us to divide all the plants into groups according to their dates of transition to the stage of floral bud formation.

Out of 96 guar lines in the experiment, only 82 successfully passed to flowering and were subjected to metabolomic profiling. Among them 30 genotypes have formed the floral buds early enough (days from the appearance of the first true leaf up to first floral bud = 41 ± 1.8, mean ± SE). For the other 52 photoperiod-sensitive genotypes, the prolonged daylight caused obstacles to the transition to a flowering program, which led to a strong delay in the formation of floral buds (95 ± 1.7, mean ± SE) [4]. We investigated metabolomic profiles of tissues of the third true leaves for plants from the two contrast groups of «early flowering» and «delayed flowering» guar genotypes (Additional file 1).

### GC-MS-metabolomic analysis of the early and delayed flowering guar genotypes

GC-MS analysis was conducted for 82 guar genotypes, each genotype in 4–6 biological replications. In order to get an insight into the technical reproducibility, at least 3 technical replicates for 3 biological replications of each line were examined. In total, 244 valid peaks were detected and semi quantified for the whole population. Based on GMD and NIST library 105 metabolites were identified including amino acids, sugars, glycosides and polyols, flavonoids, fatty acids and organic acids.

First, we checked whether the concentration of metabolites does not vary significantly among biological replications of the same sample using the relative standard deviation (RSD) approach. The value of RSD between biological replications of each line in early flowering group as well as in delayed flowering group did not exceed 20% (Additional file 2: Table S1, Fig. S1). Thus, the biological variability seen between genetically identical plants grown under identical conditions in our experiment was comparable, or even minor with those reported earlier (e.g. [5]). The technical replicates showed even lower variation: the mean of RSD, estimated for 244 metabolites, was 9% ± 5%, confirming that variability due to the methodology of experiment is minor compared to biological differences.

**Fig. 1:**
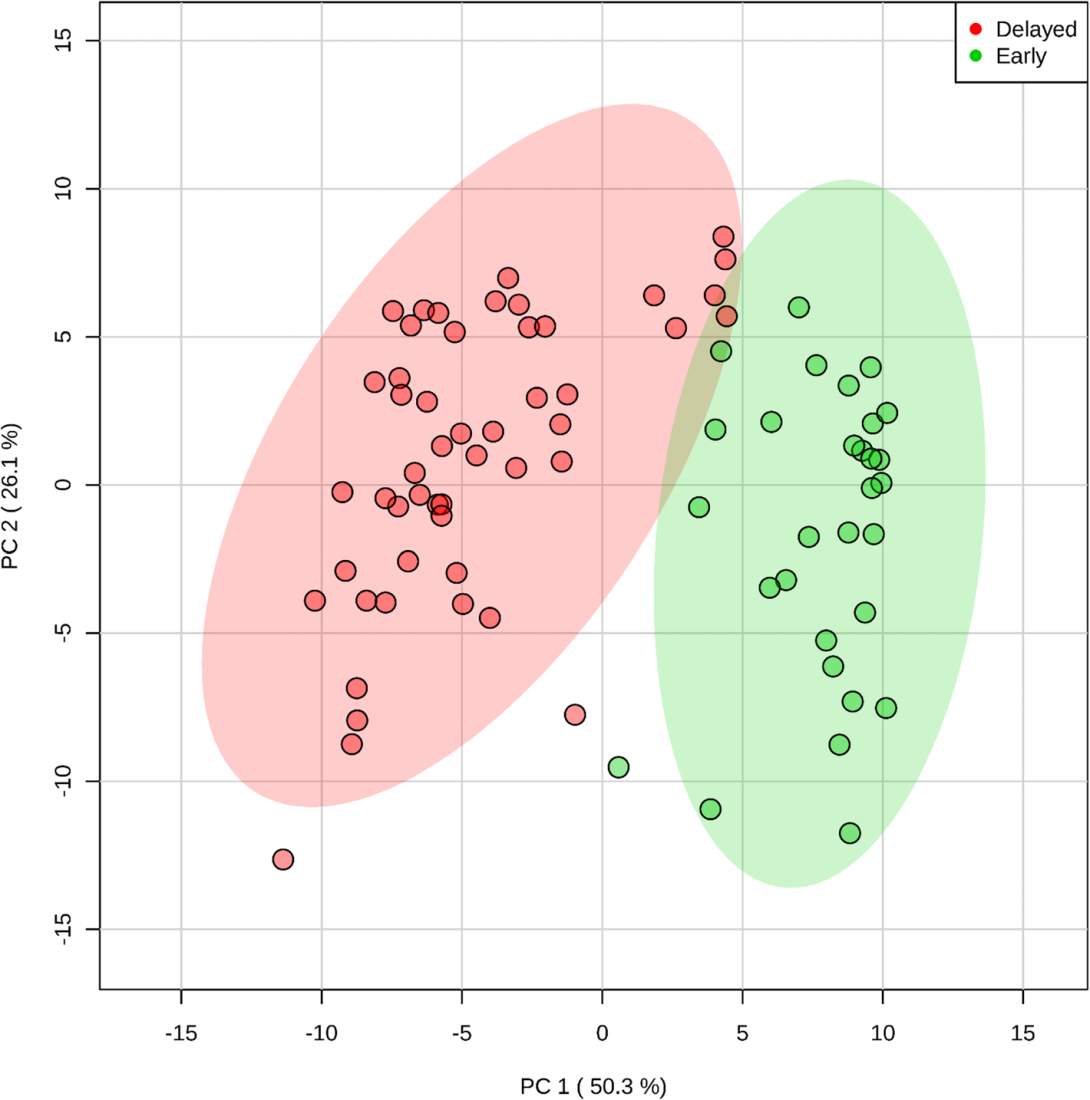
The PCA score plot based on concentrations of 244 metabolites in the sample of 82 guar genotypes. Two groups of «early flowering» and «delayed flowering» genotypes are marked by green and red correspondingly

The PCA (principal component analysis) score plot of the 244 metabolic profiles showed two clearly separated clusters of 30 early and 52 delayed flowering plants (Fig. 1). The first component, responsible for the splitting of the whole sample of 82 genotypes into two groups, explains 50.3% of variability.

**Fig. 2:**
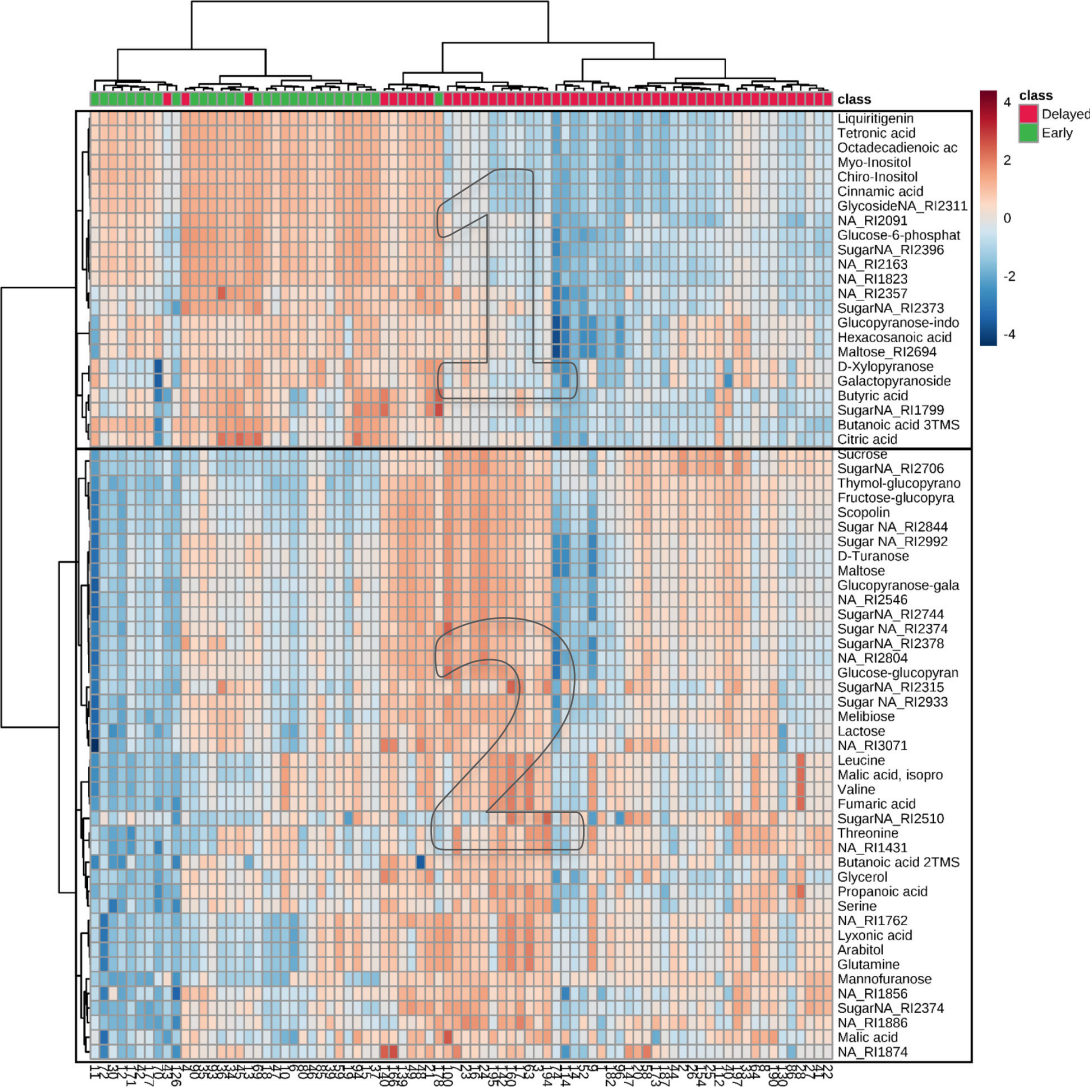
Heatmap of 65 metabolites, that were significantly different in concentrations between early (green) and delayed flowering (red) plants. Colors in each row reflect logarithm of ratio of the concentration of a metabolite in the particular genotype to the concentration of the metabolite averaged across the whole sample of 82 genotypes. The light blue boxes indicate the concentration of metabolites that are is less than the mean, and the red boxes denote concentration values that are greater than mean. The darker the color is, the larger the difference there is from the mean value

To define the metabolites that make the most significant contribution to the differentiation of guar genotypes with early and delayed flowering, we performed the t-test, which revealed 65 key metabolites (FDR value < 0.01), the concentration of those varied significantly between the two groups. Next, we cluster 82 guar genotypes according to the 65 metabolite profiles using a heatmap. As expected, the heatmap revealed that the «early flowering» and «delayed flowering» genotypes were assigned to two separated clusters (Fig. 2). There were few exceptions: genotypes with ID 4, 13, 43 previously recognized as “delayed flowering plants” were placed within the early flowering group. In fact, ID 43 metabolome profile looks identical to those in delayed flowering group (Fig. 2). Plants with ID 4 and ID 13 were slightly affected by pathogens after picking sample leaves, so they could be phenotyped incorrectly due to missed first floral buds.

Several metabolites with the higher relative concentration were identified in the early flowering genotypes, among them 2 polyols, 8 sugars, 2 glycosides, 5 organic acids, 1 flavonoid, 1 fatty acid and 4 unidentified molecules (Fig. 2, cluster 1). Other group of metabolites showed the higher relative concentration in plants with delayed flowering (2 polyols, 20 sugars, 1 glycosides, 6 organic acids, 5 amino acids, 8 unidentified) (Fig. 2, cluster 2). The detailed information about the 65 metabolites, that were significantly different in concentrations between early and delayed flowering plants is shown in Additional file 3.

Remarkably, not only genotypes that showed clear phenotypic distinction were recognized by the clustering approach, but also metabolites that belong to the same metabolite class showed the correlated variation on the heatmap. For example, the concentration of amino acids (glutamine, threonine, valine, leucine, serine) varied correspondingly in the sample of guar plants. There are also at least two clusters that combined only sugar metabolic profiles (Fig. 2).

**Fig. 3:**
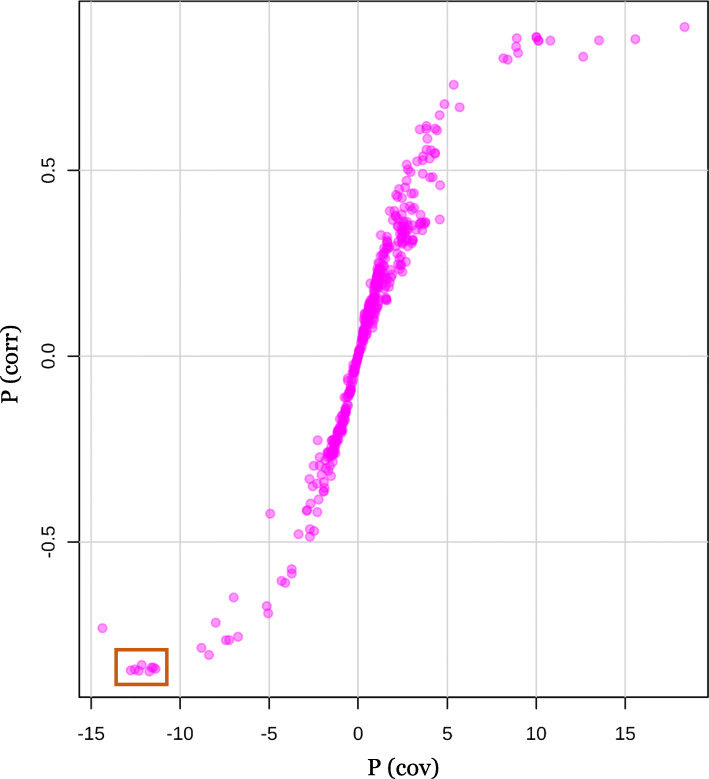
S-plot with 7 highlighted potential biomarkers discriminating metabolomes of guar plants with early and delayed onset of flowering. The x-axis, p (cov), in figure is a visualization of the contribution (covariance) to the module variables, and the y-axis, p(corr), in figure is a visualization og the reliability (correlation) of the module

Next, an S-plot was generated to further identify the statistically significant and potentially biochemically significant metabolites (Fig. 3). On the left-hand side of the S-plot, 7 metabolites with strong model contribution and high statistical reliability are highlighted as potential biomarkers associated with the rapid transition to flowering of guar plants: chiro-inositol (6TMS (Trimethylsilyl)) RI 1953 (p _cov_ = − 12.56, p_corr_ = − 0.85), myo-inositol (6TMS) RI 2088 (p _cov_ = − 11.97, p_corr_ = − 0.84), unidentified glycoside RI 2311 (p _cov_ = − 13.03, p_corr_ = − 0.85), tetronic acid (TMS) RI 2115 (p _cov_ = − 11.74, p_corr_ = − 0.84), cinnamic acid, 3,4-dihydroxy (3TMS) RI 2134 (p _cov_ = − 12.80, p_corr_ = − 0.84), unidentified metabolite RI 2358 (p _cov_ = − 12.41, p_corr_ = − 0.83), liquiritigenin RI 2437 (p _cov_ = − 11.62, p_corr_ = − 0.84). Those molecules contributed mostly to the metabolome’s discriminations between early and delayed flowering guar plants growing under stressful conditions of prolonged photoperiod.

**Fig. 4:**
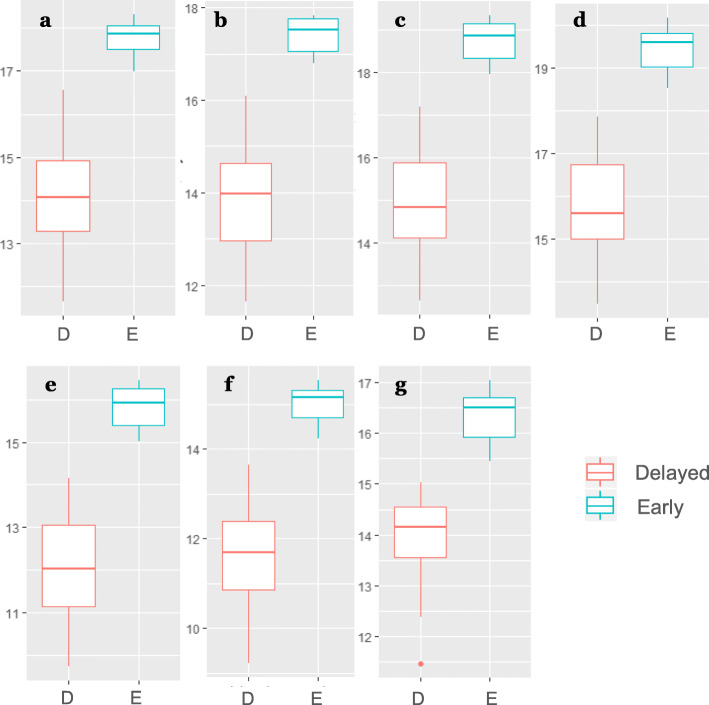
The boxplot of log normalized relative concentration of biomarkers associated with the rapid transition to flowering in guar plants, identified by S-plot. The green and red bars represent group of early (E) and delayed (D) flowering genotypes respectively. Log normalized relative concentration of the two groups of: a – chiro-inositol RI 1953; b - myo-inositol RI 2088; unidentified glycoside RI 2311; d - tetronic acid RI 2115; e - cinnamic acid, 3,4-dihydroxy RI 2134; f – liquiritigenin RI 2437; g - unidentified metabolite RI 2364

Figure 4 demonstrates Log normalized relative concentration of 7 key metabolites in groups of early and delayed flowering guar genotypes. Noticeably, all the 7 potential biomarkers have the significantly higher concentration in leaf tissues of the plants that are ready for flowering (the early flowering genotypes), suggesting the activation of the certain biochemical pathways preceding (or accompanying) the onset of flowering. Thus, a high concentration of these key molecules in the tissue of the third leaf of the guar plant indicates the upcoming flowering, while a low concentration of the molecules in these tissues means a delay in flowering, at least for the next few weeks.

## Discussion

Metabolome profiling can be employed for the detection of the key molecules and molecular mechanisms, that underlie the phenotype response to the biotic and abiotic stresses [22, 23]. When metabolome changes are investigated as a response to the stressful environmental conditions, it becomes possible both to compare the metabolite reaction of different genotypes and to understand the basics of plasticity and adaptation of the genotype to the particular stressor [22, 24, 25].

Photoperiod is the one of the most important biological factors regulating the development of plants. Changes of the daylight length serves as a signal for initiating various reactions in a plant organism, including flowering or the cessation of vegetation in the end of growing season. The metabolic changes that occur when plants grow at different daylight hours have been investigated by Goodacre et al. [26]. Using ESI-MS profiling of *Pharbitis* leaves extracts followed by discriminant analysis, the authors showed the ability to recognize plants that were grown at different photoperiods by their metabolic profiles.

In our study the metabolic response of different guar genotypes to the stress factor – prolonged photoperiod that impedes the transition to flowering in this short-day plant species – was investigated. We revealed that various guar genotypes differentiating by their photoperiod sensitivity, segregated into the early and delayed flowering groups and showed distinct metabolomic profiles. Finally, we were able to describe the metabolic landscape that accompany the timely flowering in early flowering guar genotypes.

Although the metabolomic profiling using GC Mass Spectrometry (GC-MS) does not allow to detect the entire set of metabolites presented in the examined leaf tissue [27], at least 65 metabolites were detected showing significantly different concentrations in leaves of the early and delayed flowering guar genotypes. Among them 7 key molecules with the highest concentrations in leaves of early flowering plants could be used as biomarkers for searching guar genotypes that can switch to flowering in time even under stressful conditions of a long photoperiod. That corresponds to the previous reports that diagnostics of a specific biological state of an organism is one of the greatest possibilities provided by metabolomic profiling [28].

Of the 7 key metabolites, the increased concentrations of which in leaf tissues of guar plants indicate the upcoming flowering, there were two inositol isomers. Inositol and its derivatives are crucial for development and signaling in plants, performing essential function as either metabolic mediators or participating in various signaling pathways in response to stress, hormones, and nutrients, by transcriptional regulation of the stimuli-responsive genes [29]. Myo-inositol was reported as a central component in plant cellular processes including signal transduction, stress response, cell wall biogenesis, growth regulation, osmo-tolerance, membrane trafficking [30]. Important role of inositol for the early stage of embryogenesis in plants was also described. Hence, in *Arabidopsis thaliana*, *RNA-i* induced mutations of *myo*-inositol phosphate synthase (MIPS) - the key gene for inositol biosynthesis – lead to embryo abortion [31]. Since guar is self-pollinated plant, and embryogenesis often begins in unopened flower [32] it can be assumed that a sufficient concentration of inositol in the plant tissues is a prerequisite for the floral bud formation, since the early embryo will require a guaranteed initial inositol supply for its normal development.

One of the key metabolite was attributed to flavanone liquiritigenin (Additional file 3). For legumes, up to several tens of different flavonoids were reported [33, 34], among them dihydroxyflavanone liquiritigenin was isolated from *Glycyrrhizae uralensis* Fisch. ex DC e.g. [35, 36]. Flavonoids have been recently suggested as effective endogenous regulators of auxin movement, thus behaving as developmental regulators in plants [37]. Therefore, we can assume the role of liquiquirithigenin in stress-induced morphogenic reactions of guar plants.

The detected 65 metabolites, which are highly important for transition to flowering in guar, combine 5 amino acids, 11 organic acids, 28 sugars, 3 glycosides, 4 polyols, 1 flavonoid, 1 fatty acid and 12 unknown metabolites. Significant differences (FDR value < 0.01) in their concentrations between early and delayed flowering plants affect several pathways according to the KEGG database: valine, leucine and isoleucine biosynthesis; glycerolipid metabolism; glycine, serine and threonine metabolism; D-glutamine and D-glutamate metabolism; N-, O-glycan biosynthesis; gluconeogenesis; pentose phosphate pathway; nucleotide sugar biosynthesis, galactose degradation; glycolysis; ascorbate biosynthesis; trehalose biosynthesis; galactose degradation; glycogen biosynthesis; inositol phosphate metabolism; glycosylphosphatidylinositol (GPI)-anchor biosynthesis; phosphatidylinositol signaling system; trans-cinnamate degradation and linoleic acid metabolism.

Since the metabolome is the end result of numerous biochemical pathways, one should consider that the effective running of these pathways depends on the corresponding enzymes, which, in turn, are encoded by genes. Metabolites’ variation can be considered as the inherited trait, thus, metabolomic profiling is employed in genetic studies [38–40]. There are several reports about the QTL mapping of genes responsible for metabolites’ variation [41–43]. Likewise, our study opens up the potential for searching genetic loci associated with guar plant flowering via detecting of genes involved in the biosynthesis of the key identified metabolites. This becomes possible due to combining the capabilities of GC-MS with the latest advances in bioinformatics [22, 23], which provide additional opportunities for functional genetics.

## Conclusion

The metabolomic landscape accompanying transition to flowering in guar was firstly described. Under the stressful long daylight (17–18 h) conditions those plants which are ready to switch to flowering show the metabolome profile different from that in plants with delayed flowering in concentrations of at least 65 metabolites. In particular, the onset of flowering in guar is associated with a dramatic increase of concentrations of 7 key metabolites: chiro-inositol (RI 1953), myo-inositol (RI 2088), tetronic acid (RI 2115), cinnamic acid, 3,4-dihydroxy (RI 2134), unidentified glycoside (2311), liquiritigenin (RI 2437) and unidentified metabolite (RI 2364). The higher concentrations of those metabolites can be detected in tissues of the third true leaf – the developmental stage that precede first floral bud appearance. These molecules can be employed as biomarkers for the rapid screening of breeding material to reveal the potentially early flowering guar genotypes on a stage of the third true leaf. That could assist breeding of new guar varieties that are more adapted for cultivation of the short-day species in the countries with prolonged photoperiod.

## Methods

### Study design and sample collection

96 guar genotypes of different geographic origin from the VIR collection were selected for the study. In this sample the local varieties from India, known cultivars from USA (Kinman, Lewis, Santa Cruz), as well as recently developed varieties from Russia (Vavilovskij 130, Vector, Sinus) were presented (Additional file 4). In 2017 the selected 96 guar genotypes were propagated in the Kuban experimental station of VIR (Krasnodar, Russia). Seed reproduction was collected from the each of 96 genotypes individually. In 2018, 8 seeds of each genotype were sown in soil in pots in the greenhouse of Pushkin branch of VIR (St. Petersburg region, 59°53′39″N) where the plants were grown in the equal conditions of light, humidity and temperature (Additional file 5). During the experiment, the plants were not exposed to any agro-biological treatments.

For all the plants the date of appearance of seedlings (germination), the date of appearance of the first true leaf and the date of appearance of the first flower were recorded, after that the rate of the transition to the generative phase were calculated for each genotype. As previously reported, the genotype was recorded as “early flowering” if it turned to flowering within 41 ± 1,8 from the first true leaf appearance. Correspondingly, a genotype was assigned to the “delayed flowering” group if it switched to flowering late (after 95 ± 1,7 days) [4].

Since each of 96 genotype was represented by 8 plants, for GS-MC-metabolomic analysis the third true leaf were separately collected from up 4 to 6 plants of each genotype as biological replications. The sample picking was carried out in June, 2018 (evening time). The leaves were immediately weighed and frozen in liquid nitrogen. The storage of samples was carried out at a temperature of - 80 °С.

### Extraction of compounds and metabolite derivatization

The metabolites of guar leaves were extracted after freezing in cold methanol in 1.5 mL Eppendorf type microtubes (SSI, USA) during 1 h at + 4 °С [27]. The extract solution was transferred to clear Eppendorf microtubes and evaporated using the vacuum concentrator (Labconco, USA).

Derivatization was carried out by silylation method. For this purpose, dry metabolites were dissolved in 50 μl pyridine and 20 μl internal standard tricosane (nC23, Sigma) in pyridine solution (1 μg/μl). Silylation was carried out using 50 μl N,O-Bis (trimethylsilyl) trifluoroacetamide (BSTFA, Sigma).

### Metabolite identification by GC-MS

GC-MS analysis of the samples was performed with the gas chromatograph system (Agilent 6850, USA) in cooperation with mass-spectrometer (Agilent 5975B, USA). The system used a DB-5HT capillary column coated with 5%cross-linked diphenyl (30 m × 250 μm inner diameter, 0.25 μm film thickness; Agilent J&W, USA). 0.8 μm aliquot of the sample was added in splitless mode. Helium was used as the carrier gas. The flow of the front inlet purge was 1 mL/min. The original temperature was set at 70 °С. The temperature was increased from 70°С to 340°С at a speed of 4 °C/min. Temperature 250°С was used for the injection. The full-scan mode of the mass spectrometry data was 50 m/z – 800 m/z at a rate of 2 spectra scan per second. The chromatogram recording was performed on the signal of the total ion current by Agilent ChemStation soft.

The peak detection and measurement of integrated area of peaks carry out by UniChrome 5.0.19.1162 (www.unichrom.com). The calculation of relative concentration on the weight of sample and concentration internal standard tricosane (1 μg/μl) was performed by methods of semi quantitative analysis.

For GC-MS-analysis, in average, 5 replicates of each genotype were used. As the result, a minimum 3 good-quality chromatogram were obtained for each genotype. The calculating of concentration value for each detected metabolite was performed by averaging of all reps available, taking into account a value of relative standard deviation (RSD) [5, 44].

Identification of metabolites was performed with Automated Mass Spectral Deconvolution and Identification System AMDIS 32 (http://www.amdis.net/) using library NIST/EPA/NIH 08 Mass Spectral Library (http://www.nist.gov/srd) and database of mass spectrometric information, created at the Komarov Botanical Institute. Then the results (10 largest peaks and Retention Index (RI)) were verified by comparison with database GMD, Golm Metabolome Database (http://gmd.mpimp-golm.mpg.de/analysisinput.aspx). The metabolite was considered identified if Match factor values exceeded threshold 700.

### Statistical analysis of differentially expressed metabolites in groups

The multivariate statistical processing of metabolomic data was carried out using online analysis platform MetaboAnalyst 4.0 (http://www.metaboanalyst.ca) [45]. Data have been subjected to the log transformation (generalized logarithm transformation or glog).

One-way ANOVA (t-test) analysis were used to identify important metabolites discriminating two groups. When FDR *p*-value was less than 0.01, a metabolite was characterized as significantly different in its concentration between the groups. Multivariate analysis included hierarchical cluster analysis (Heatmap), principal component analysis (PCA) and orthogonal projections to latent structures (OPLS) with constructed S-plot for orthogonal features. Preprocessing of data for multivariate analysis included missing value estimation. Missing values were replaced by the lowest values (half of the minimum positive value in the original data). Data filtering and data scaling was not performed.

The Heatmap provides intuitive visualization of a data table of concentration of metabolites in different samples. Each colored cell on the map corresponds to a concentration value in the data table, with samples in rows and features/compounds in columns: the redder - the higher the logarithm of concentration. The blue color - the lower the concentration logarithm. Data clustering was performed based on the Euclidean distance estimation using Ward Clustering algorithm.

Using the PCA analysis method, the two-dimensional model was constructed confirming the differences between groups and displaying the general similarity and difference between samples. An S-plot [46] was further generated to identify statistically significant metabolites discriminating early and late flowering plants, i.e. showing the highly significant negative correlation.

## Supplementary information

**Additional File 1.** The scheme of collecting of the biological material for metabolome profiling experiments.

**Additional File 2. **Biological variation (%, RSD) of 244 metabolite concentrations among 82 genotypes in groups of early and delayed flowering plants. **Table S1.** Biological variation (%, RSD) of 244 metabolite concentrations among 82 genotypes in groups of early and delayed flowering plants. **Fig. S1.** Biological variation (%, RSD) of concentrations mean of 244 metabolites among 82 genotypes in groups of early and delayed flowering plants

**Additional File 3.** The 65 metabolites, which significantly differ in their concentrations between groups of early and delayed flowering plants

**Additional File 4.** The geographical location and accession numbers of the VIR Collection of guar genotypes

**Additional File 5.** The conditions of day light, humidity and temperature of the greenhouse of Pushkin branch of VIR

## Data Availability

Supporting data are included as additional files. The all metabolomics data of third leafs early and delayed flowering plants have been submitted to MetaboLights (EMBI-EBI) under accession number MTBLS1589.

## Abbreviations

GC-MS: Gas Chromatography –Mass Spectrometry
SE: Standard Error of Mean;
TMS: Trimethylsilyl
GMD: Golm Metabolome Database
NIST: National Institute of Standards and Technology
RSD: Relative Standard Deviation
PCA: Principal Component Analysis
OPLS: Orthogonal projections to latent structures
