# Supplementary material for: Key metabolites associated with the onset of flowering of guar genotypes (Cyamopsis tetragonoloba (L.) Taub)
Source: BMC Plant Biol. 2020 Oct 14;20(Suppl 1):291. doi: 10.1186/s12870-020-02498-x (PMC7557002; doi:10.1186/s12870-020-02498-x)

The scheme of collecting of the biological material for metabolome profiling experiments

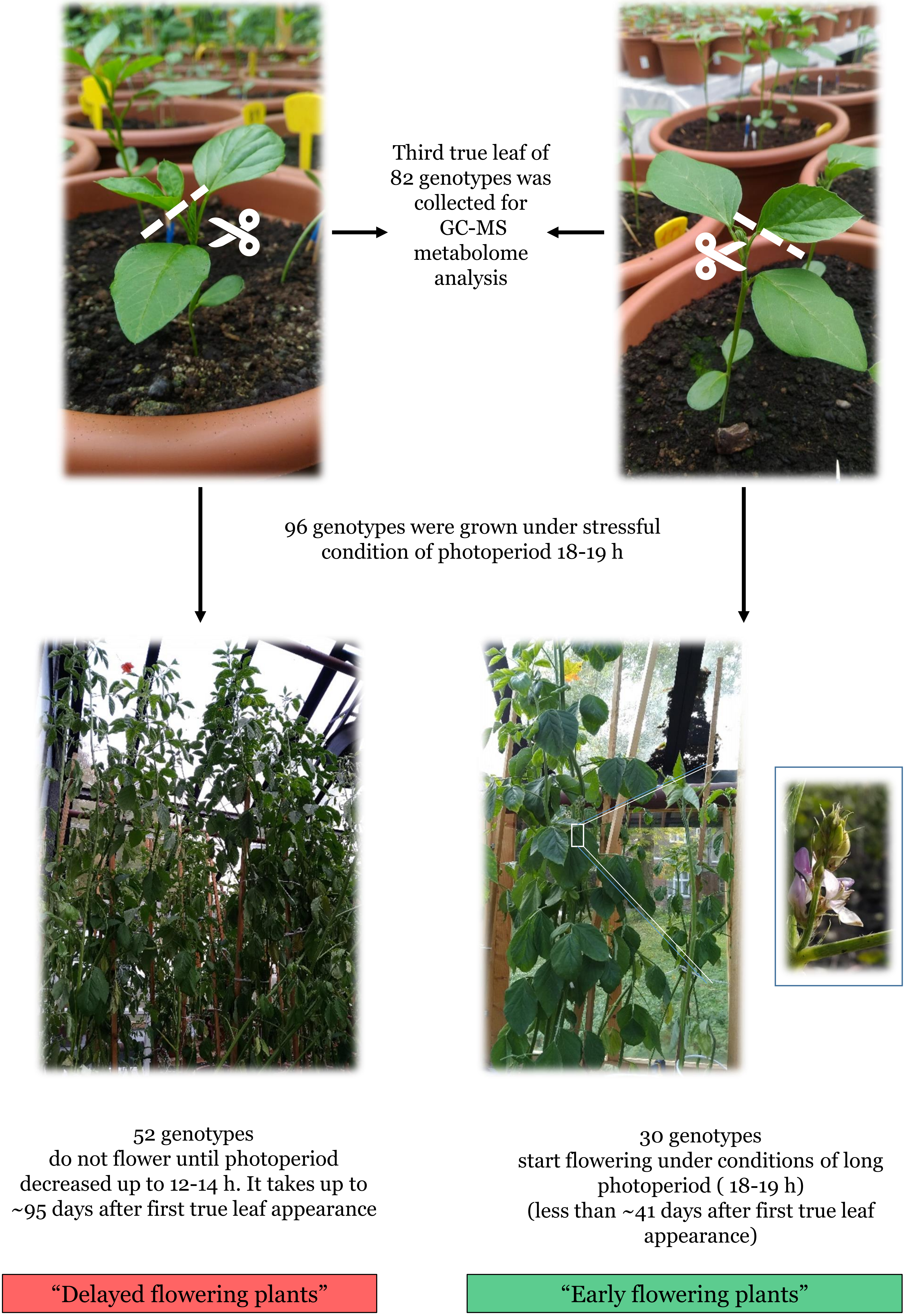

Supplement: Supplementary file 1 — Additional File 1. The scheme of collecting of the biological material for metabolome profiling experiments. [file 12870_2020_2498_MOESM1_ESM.pdf]
