# Supplementary material for: Key metabolites associated with the onset of flowering of guar genotypes (Cyamopsis tetragonoloba (L.) Taub)
Source: BMC Plant Biol. 2020 Oct 14;20(Suppl 1):291. doi: 10.1186/s12870-020-02498-x (PMC7557002; doi:10.1186/s12870-020-02498-x)

Table S1. Biological variation (%, RSD) of 244 metabolite concentrations among 82 genotypes in groups of early and delayed flowering plants

Metabolite[1]	Early		Delayed	
	RSD	Err std	RSD	Err std
1105	18	2.6	21	2.3
1176	16	1.8	17	1.9
1294	15	2.0	19	2.0
1350	16	1.7	22	2.1
1358	13	2.2	18	2.0
1357	15	1.9	16	1.3
1383	13	2.0	19	2.1
1495	12	1.8	17	2.0
1503	14	1.9	19	2.4
1522	14	2.1	18	2.2
1528	18	3.0	24	2.5
1577	18	2.1	22	2.0
1620	14	1.9	18	2.2
1274	12	1.7	16	2.1
1167	13	1.9	16	2.0
1204	18	2.0	18	1.7
1213	16	2.1	18	2.1
1262	13	2.9	18	2.5
1269	12	1.7	17	1.9
1312	14	1.8	18	2.2
1671	20	2.6	26	2.2
1764	14	2.3	18	2.4
1803	9	2.5	14	2.3
1813	17	2.1	19	2.2
1821	15	1.9	21	2.5
1835	14	2.1	20	2.3
1849	13	1.7	18	2.2
1858	13	2.0	16	1.9
1888	11	2.2	18	2.1
1890	18	2.1	21	2.4
1930	16	2.7	20	2.2
1953	13	1.7	21	2.4
1982	16	2.4	20	2.7
2088	20	2.6	27	2.6
2176	18	2.1	22	2.2
2305	15	1.4	19	1.9
2358	19	2.3	17	1.6
2643	11	2.1	ND	
2637	ND[2]		38	16.2
3251	16	1.8	19	2.0
3311	17	1.9	20	2.1
3129	13	3.3	15	2.4
1742	14	2.6	17	1.6
1738	12	1.7	17	2.1
1782	15	2.2	19	2.6
1294	16	2.6	19	2.2

1505	12	1.9	18	2.1
1511	11	1.7	16	2.2
1522	5	1.7	10	4.0
1546	2	2.1	ND	
1558	14	1.8	19	2.1
1576	13	2.2	18	1.9
1589	15	5.2	19	3.9
1622	16	2.5	20	2.5
1691	12	2.7	12	2.3
1720	1	0.7	16	7.9
1741	7	2.2	11	2.4
1746	ND		19	12.4
1770	18	2.6	22	2.1
1774	11	6.0	8	1.8
1796	2	0.1	17	4.9
1799	12	2.4	19	2.5
1830	19	3.4	15	3.2
1839	16	3.6	20	3.1
1868	15	3.1	17	2.7
1871	13	2.0	16	1.9
1892	8	1.9	15	7.5
1900	17	3.7	19	3.3
1906	14	3.7	18	3.6
1926	11	3.2	16	2.5
1931	8	2.2	10	5.7
1940	13	2.6	18	2.6
1957	15	2.0	20	2.5
1965	17	2.6	20	2.5
1979	13	2.6	15	2.9
1988	21	2.5	19	1.6
1997	19	2.4	22	2.4
2042	12	1.7	17	2.0
2059	11	2.5	20	3.0
2091	10	4.5	9	2.0
2196	3	1.4	ND	
2204	6	2.1	ND	
2210	5	1.1	12	3.2
2311	19	2.0	19	2.2
2520	20	2.9	19	2.9
2586	19	2.5	19	1.8
2632	14	1.9	16	2.1
2643	20	2.2	22	2.3
2691	18	2.7	18	2.2
1325	10	1.7	17	2.2
1364	7	2.7	4	1.3
1365	10	1.5	11	1.9
1386	13	2.3	16	2.0
1393	12	1.4	15	1.8
1414	17	2.1	20	2.1
1420	13	2.9	10	2.3

1430	12	1.7	15	2.1
1441	16	2.0	19	2.5
1448	9	1.6	11	1.9
1463	13	1.9	16	1.8
1466	13	2.0	16	2.0
1477	10	1.6	15	2.3
1540	13	1.8	16	2.2
1555	4	1.1	15	11.2
1567	12	1.7	14	1.6
1596	14	1.4	15	1.7
1597	13	3.8	15	2.0
1612	11	1.3	14	1.8
1641	12	1.7	14	1.7
1661	14	1.7	15	1.9
1676	12	6.4	11	3.0
1699	13	2.5	17	2.3
1706	12	1.7	15	1.7
1713	21	13.8	24	23.9
1717	14	1.8	15	1.9
1729	10	1.0	12	1.9
1743	9	1.8	11	1.7
1747	14	2.1	17	2.2
1770	18	2.6	22	2.1
2059	14	2.0	16	1.7
2802	18	2.2	21	2.1
2614	19	2.2	20	2.0
3326	11	1.7	15	2.0
1528	14	1.8	18	2.1
1759	14	2.0	16	2.0
1786	19	2.1	20	2.1
2112	16	2.3	15	2.0
2197	18	2.3	18	1.5
2205	8	0.9	11	1.5
1166	15	5.0	25	4.1
1219	ND		10	3.2
1251	11	5.5	22	3.6
1904	ND		25	0.9
2066	19	4.0	11	1.8
2134	10	1.9	16	2.3
2243	16	1.6	19	2.0
2389	21	5.4	25	5.1
2547	16	2.1	16	1.9
2559	14	4.7	16	3.4
2608	9	2.9	18	3.8
2965	13	2.4	19	2.1
3127	14	1.8	16	1.8
1675	15	1.7	17	2.2
1720	11	1.6	14	1.7
1641	8	6.0	ND	
1645	11	3.2	6	0.9

1784	4	0.9	15	3.3
1870	13	2.4	14	2.1
1896	8	1.8	8	1.7
2507	16	2.0	19	2.0
2626	15	2.8	19	2.0
2680	15	2.3	13	1.5
1843	17	4.0	15	1.8
2190	16	2.1	19	2.0
2585	10	2.3	10	2.0
2935	14	1.6	17	2.0
2979	13	5.3	11	2.0
3001	13	1.9	16	1.8
1547	11	1.6	12	1.5
2437	13	2.2	21	3.1
1117	11	4.2	16	2.3
1515	ND		54	5.2
1693	10	1.8	13	1.5
2053	12	2.2	16	2.4
2216	8	1.5	15	2.5
2466	13	2.7	15	2.3
1363	7	2.6	8	1.9
1929	5	2.6	26	4.1
2203	3	0.3	9	2.7
1058	11	1.8	13	1.6
1064	14	3.7	12	2.1
931	13	2.3	ND	
931	ND		11	2.1
938	16	1.9	16	1.8
939	12	2.1	13	1.7
951	14	2.8	15	1.6
958	8	2.1	11	1.9
961	14	2.2	15	2.7
975	10	1.8	16	4.8
975	6	2.3	ND	
992	8	0.7	13	1.7
1002	14	2.5	9	2.0
1002	ND		5	1.7
1019	10	2.6	11	2.8
1028	7	1.5	11	1.8
1030	16	2.1	21	2.3
1035	7	1.1	13	2.7
1036	5	1.3	10	2.2
1074	12	5.2	14	3.1
1076	15	4.6	15	3.0
1085	8	1.3	13	2.3
1091	14	2.5	12	2.1
1142	11	4.4	10	3.8
1188	10	1.9	13	5.2
1189	ND		13	4.6
1235	10	3.4	12	1.9

1245	ND		9	2.6
1250	14	3.2	10	2.6
1269	11	2.2	13	1.7
1289	11	1.8	15	1.8
1292	16	2.3	21	2.2
1300	ND		10	8.3
1304	9	2.2	9	2.1
1304	11	1.8	15	2.5
1339	10	2.8	9	3.2
1367	9	0.9	10	6.3
1367	6	1.7	10	1.8
1372	9	2.0	12	1.5
1386	11	1.9	14	1.5
1390	14	2.8	12	1.6
1399	20	11.8	15	1.3
1410	9	2.0	10	1.5
1448	4	2.3	ND	
1461	3	0.8	12	4.4
2182	3	1.5	12	2.2
2530	8	1.8	15	2.8
2701	ND		19	5.3
2643	14	2.8	17	2.3
2009	13	1.8	14	1.8
1296	10	3.6	7	1.6
1446	8	2.5	11	2.1
1795	8	1.9	10	2.1
1905	16	2.8	19	2.2
1892	14	1.9	16	2.1
1931	6	1.6	9	1.5
1960	14	3.5	13	2.1
1874	12	2.2	15	3.0
1879	14	2.9	11	1.8
1826	11	2.7	ND	
1940	13	2.6	18	2.6
1941	ND		13	3.8
1961	ND		23	7.6
1984	12	10.0	ND	
1985	ND		14	13.1
1987	ND		12	3.5
1989	ND		15	2.5
2208	12	4.3	12	3.3
2373	13	2.4	15	2.6
2388	15	2.1	16	2.1
2651	18	3.5	ND	
1952	10	1.2	13	2.1
2096	9	1.6	12	1.4
2790	10	1.5	9	1.9
2096	14	1.4	19	1.9

[1]metabolites shown via RI index

[2]ND - not detected

Fig S1. Biological variation (%, RSD) of concentrations mean of 244 metabolites among 82 genotypes in groups of early and delayed flowering plants

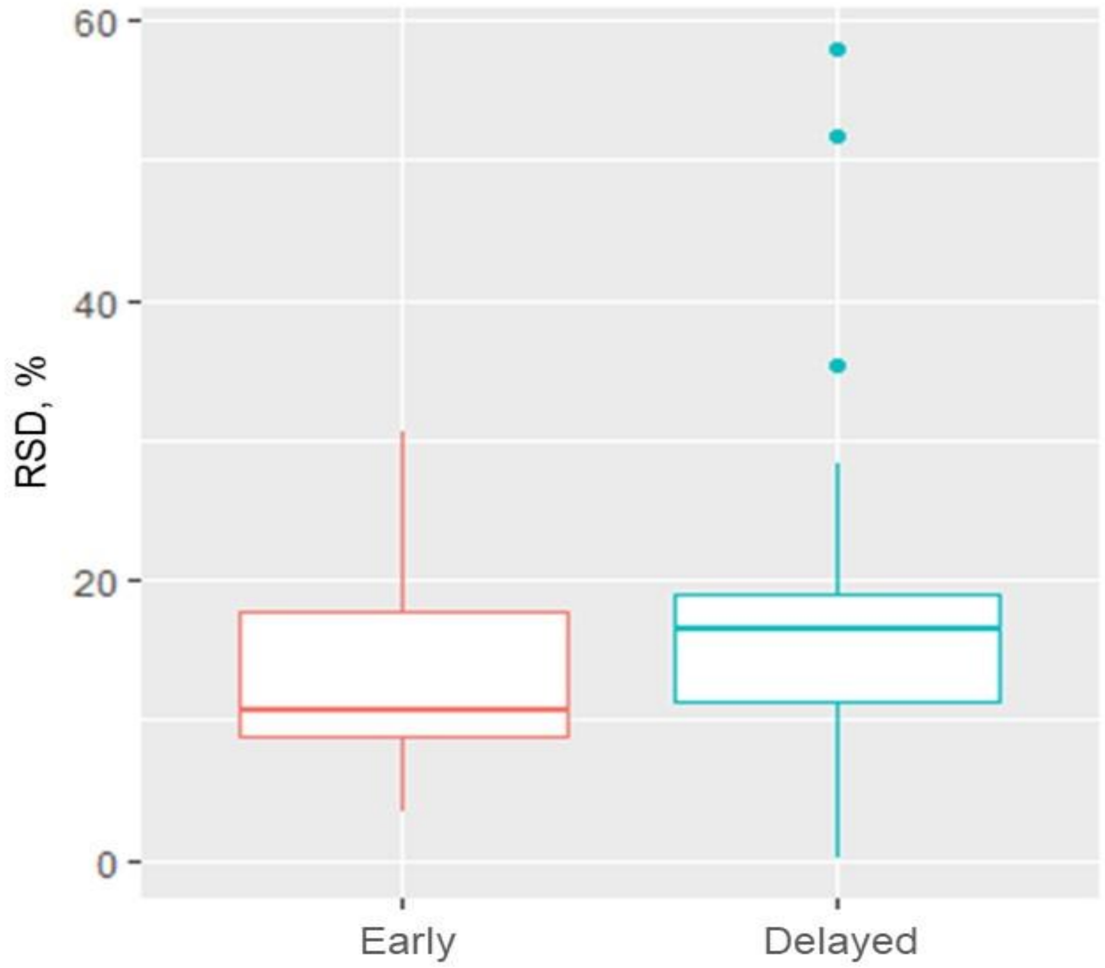

Supplement: Supplementary file 2 — Additional File 2. Biological variation (%, RSD) of 244 metabolite concentrations among 82 genotypes in groups of early and delayed flowering plants. Table S1. Biological variation (%, RSD) of 244 metabolite concentrations among 82 genotypes in groups of early and delayed flowering plants. Fig. S1. Biological variation (%, RSD) of concentrations mean of 244 metabolites among 82 genotypes in groups of early and delayed flowering plants [file 12870_2020_2498_MOESM2_ESM.pdf]
