# Supplementary material for: Key metabolites associated with the onset of flowering of guar genotypes (Cyamopsis tetragonoloba (L.) Taub)
Source: BMC Plant Biol. 2020 Oct 14;20(Suppl 1):291. doi: 10.1186/s12870-020-02498-x (PMC7557002; doi:10.1186/s12870-020-02498-x)
Supplement: Supplementary file 3 — Additional File 3. The 65 metabolites, which significantly differ in their concentrations between groups of early and delayed flowering plants [file 12870_2020_2498_MOESM3_ESM.pdf]

The 65 metabolites, which significantly differ in their concentrations between groups of early and delayed flowering plants

№	Metabolite	p.value	-lg(p)	FDR	Match	RI	RT (min)
1	Valine (2TMS)	*[2]	4.9476	*	888	1210	12.7
2	Leucine (2TMS)	*	5.0271	*	893	1266	14.56
3	Glycerol (3TMS)	*	4.7319	*	943	1272	14.74
4	Butanoic acid, 4-amino- (2TMS)	*	8.4111	*	795	1294	15.96
5	Glyceric acid (3TMS)	*	5.1012	*	924	1325	16.47
6	Fumaric acid (2TMS)	*	4.3585	*	939	1350	17.19
7	Serine (3TMS)	*	7.4173	*	945	1356	17.48
8	Threonine (3TMS)	*	8.0893	*	928	1381	18.27
9	NA[1]	*	7.4123	*	<700	1431	19.86
10	Malic acid (3TMS)	*	5.009	*	955	1492	21.75
11	Butanoic acid, 4-amino- (3TMS)	*	11.456	*	837	1525	22.72
12	Malic acid, 3-isopropyl-, threo- (3TMS)	*	4.6907	*	780	1567	23.96
13	Arabitol (5TMS)	*	7.7387	*	903	1720	28.34
14	Lyxonic acid (5TMS)	*	8.0682	*	791	1747	29.04
15	NA	*	9.2616	*	<700	1762	29.44
16	Glutamine, DL- (3TMS)	*	7.6108	*	868	1770	29.68
17	NA	*	4.8582	*	<700	1780	29.94
18	SugarNA	*	5.2923	*	<700	1799	30.48
19	Citric acid (4TMS)	*	10.272	*	955	1819	30.94
20	SugarNA	*	10.294	*	<700	1823	31.56
21	NA	*	6.714	*	<700	1856	31.91
22	NA	*	4.5188	*	<700	1874	32.3
23	NA	*	8.4111	*	<700	1886	32.63
24	D-Xylopyranose (4TMS)	*	4.3174	*	<700	1888	32.7
25	α-Mannofuranose (5TMS)	*	11.451	*	<700	1896	32.97
26	Chiro-Inositol (6TMS)	*	18.098	*	756	1953	34.39
27	β-D-Galactopyranoside (4TMS)	*	7.3387	*	<700	1981	35.01
28	Myo-Inositol (6TMS)	*	16.488	*	886	2088	37.56
29	NA	*	12.079	*	<700	2091	37.67
30	Tetronic acid (TMS)	*	14.064	*	672	2115	38.15
31	Cinnamic acid, 3,4-dihydroxy (3TMS)	*	14.241	*	885	2134	38.54
32	NA	*	12.823	*	<700	2163	39.18
33	Octadecadienoic acid, n- (1TMS)	*	14.008	*	878	2213	40.33
34	GlycosideNA	*	14.606	*	<700	2311	42.53
35	SugarNA	*	6.6642	*	875	2315	42.52
36	NA	*	8.1334	*	<700	2357	43.46
37	NA	*	12.004	*	487	2358	43.45
38	Sugar NA	*	9.7115	*	<700	2373	43.75
39	Sugar NA	*	8.0369	*	<700	2374	43.75
40	Sugar NA	*	5.303	*	<700	2378	43.82
41	Glucose-6-phosphate (6TMS)	*	11.629	*	809	2389	44.05
42	SugarNA	*	12.528	*	<700	2396	44.46
43	Liquiritigenin	*	14.446	*	<700	2437	45.08
44	Sugar NA	*	6.6187	*	721	2510	46.47
45	Thymol-β-D-glucopyranoside (4TMS)	*	11.682	*	<700	2523	46.73
46	NA	*	9.8673	*	<700	2546	47.12
47	α-D-Glucopyranoside-β-D-fructofuranosyl (8TMS)	*	9.3933	*	<700	2589	47.99

48	Sucrose (8TMS)	*	11.246	*	928	2634	48.85
49	NA	*	9.7399	*	<700	2646	49.06
50	Maltose	*	9.3483	*	773	2694	49.97
51	SugarNA	*	11.208	*	<700	2706	50.17
52	D-Glucopyranose-β-D-galactopyranosyl (8TMS)	*	10.117	*	<700	2729	50.26
53	SugarNA	*	10.37	*	<700	2744	50.38
54	Scopolin (4TMS)	*	11.157	*	<700	2780	51.74
55	NA	*	5.6328	*	<700	2804	51.95
56	D-Glucose-β-D-glucopyranosyl (8TMS)	*	8.9066	*	<700	2827	52.73
57	Sugar NA	*	10.617	*	<700	2844	53.58
58	D-Turanose (6TMS)	*	4.6639	*	<700	2875	53.74
59	D-Fructose-α-D-glucopyranosyl (8TMS)	*	11.434	*	<700	2921	53.86
60	Sugar NA	*	4.9176	*	<700	2933	54.16
61	Maltose (8TMS)	*	6.1898	*	<700	2955	54.34
62	Melibiose (8TMS)	*	8.4685	*	<700	2977	54.62
63	Sugar NA	*	7.2345	*	<700	2992	55.24
64	Lactose (8TMS)	*	4.5843	*	<700	3032	55.56
65	NA	*	5.447	*	<700	3071	56.42

[1]NA - unidentified

[2] ∗ - value <0.01. High statistical significance was defined at P< 0.01. FDR < 0.01 indicated high statistical significance after multiple comparisons test
