# Supplementary material for: Key metabolites associated with the onset of flowering of guar genotypes (Cyamopsis tetragonoloba (L.) Taub)
Source: BMC Plant Biol. 2020 Oct 14;20(Suppl 1):291. doi: 10.1186/s12870-020-02498-x (PMC7557002; doi:10.1186/s12870-020-02498-x)
Supplement: Supplementary file 4 — Additional File 4. The geographical location and accession numbers of the VIR Collection of guar genotypes [file 12870_2020_2498_MOESM4_ESM.pdf]

The geographical origin and the VIR Catalogue accession number of guar genotypes analysed

№	ID guar genotypes	Guar genotype	VIR Cat.	Geographical location
1	1	local variety	8	India
2	2	local variety	11	India
3	3	local variety	15	India
4	4	local variety	22	India
5	5	local variety	24	India
6	6	local variety	28	India
7	7	local variety	28	India
8	8	local variety	29	India
9	9	local variety	33	India
10	10	local variety	64	India
11	11	local variety	66	India
12	12	local variety	54	India
13	13	local variety	60	India
14	14	local variety	60	India
15	15	local variety	64	India
16	16	local variety	64	India
17	17	local variety	69	India
18	18	local variety	69	India
19	19	local variety	70	India
20	20	local variety	71	Australia
21	21	local variety	72	India
22	22	local variety	73	Australia
23	23	local variety	74	Australia
24	24	local variety	75	India
25	25	local variety	75	India
26	26	local variety	76	India
27	27	local variety	77	India
28	28	local variety	77	India
29	29	local variety	78	India
30	30	local variety	78	India
31	31	local variety	79	India
32	32	local variety	81	India
33	33	local variety	81	India
34	34	local variety	82	India
35	35	local variety	82	India
36	36	local variety	83	India
37	37	local variety	83	India
38	38	local variety	87	India
39	39	local variety	87	India
40	40	local variety	89	India
41	41	local variety	90	India
42	42	local variety	90	India
43	43	local variety	91	India
44	44	local variety	91	India

45	45	local variety	94	India
46	46	local variety	94	India
47	47	local variety	97	India
48	48	local variety	52581	Pakistan
49	49	local variety	52581	Pakistan
50	51	local variety	52581	Pakistan
51	52	local variety	53181	India
52	53	local variety	53181	India
53	55	local variety	53181	India
54	58	local variety	53182	Pakistan
55	59	local variety	53182	Pakistan
56	61	local variety	53182	Pakistan
57	63	local variety	53182	Pakistan
58	64	local variety	52569	Pakistan
59	69	Kinman	52585	USA
60	70	Kinman	52585	USA
61	72	Kinman	52585	USA
62	74	local variety	52580	Pakistan
63	80	Sinus	52575	Russia
64	81	Sinus	52575	Russia
65	84	Vector	52574	Russia
66	85	Vector	52574	Russia
67	86	Vector	52574	Russia
68	90	Santa Cruz	52584	USA
69	93	Lewis	52586	USA
70	94	Lewis	52586	USA
71	96	Lewis	52586	USA
72	100	Vavilovskij 130	52572	Russia
73	113	local variety	52573	Pakistan
74	115	local variety	52573	Pakistan
75	126	local variety	53182	Pakistan
76	130	local variety	52580	Pakistan
77	139	local variety	52581	Pakistan
78	140	local variety	52581	Pakistan
79	142	local variety	52581	Pakistan
80	147	local variety	52581	Pakistan
81	152	Kinman	52585	USA
82	154	Kinman	52585	USA
83	159	Santa Cruz	52584	USA
84	160	Santa Cruz	52584	USA
85	166	Santa Cruz	52584	USA
86	168	Lewis	52586	USA
87	171	Lewis	52586	USA
88	173	Lewis	52586	USA
89	177	Lewis	52586	USA
90	178	Lewis	52586	USA
91	182	local variety	52589	Pakistan
92	187	local variety	52589	Pakistan

93	190	local variety	52589	Pakistan
94	194	local variety	52589	Pakistan
95	195	local variety	52589	Pakistan
96	197	local variety	52589	Pakistan
