# Supplementary material for: Key metabolites associated with the onset of flowering of guar genotypes (Cyamopsis tetragonoloba (L.) Taub)
Source: BMC Plant Biol. 2020 Oct 14;20(Suppl 1):291. doi: 10.1186/s12870-020-02498-x (PMC7557002; doi:10.1186/s12870-020-02498-x)
Supplement: Supplementary file 5 — Additional File 5. The conditions of day light, humidity and temperature of the greenhouse of Pushkin branch of VIR [file 12870_2020_2498_MOESM5_ESM.pdf]

The conditions of day light, humidity and temperature of the greenhouse of Pushkin branch of VIR

Day of experiment	Day time		Night time		Day length
	Temperature, ºC	Humidity, %	Temperature, ºC	Humidity, %	
01.06.2018	31	47	21	56	18 h 17 min
02.06.2018	33	42	20	53	18 h 20 min
03.06.2018	30	49	21	59	18 h 23 min
04.06.2018	26	60	19	65	18 h 26 min
05.06.2018	21	71	16	78	18 h 28 min
06.06.2018	22	74	18	74	18 h 32 min
07.06.2018	21	71	17	80	18 h 34 min
08.06.2018	24	65	20	85	18 h 36 min
09.06.2018	25	70	17	75	18 h 39 min
10.06.2018	26	71	19	79	18 h 40 min
11.06.2018	28	75	23	71	18 h 42 min
12.06.2018	27	64	22	84	18 h 44 min
13.06.2018	29	68	24	76	18 h 45 min
14.06.2018	33	51	25	70	18 h 47 min
15.06.2018	34	52	25	65	18 h 47 min
16.06.2018	35	45	25	63	18 h 49 min
17.06.2018	32	42	24	66	18 h 50 min
18.06.2018	34	41	25	81	18 h 50 min
19.06.2018	29	89	22	90	18 h 51 min
20.06.2018	24	80	21	91	18 h 51 min
21.06.2018	22	74	20	75	18 h 51 min
22.06.2018	21	65	18	51	18 h 51 min
23.06.2018	19	51	18	51	18 h 50 min
24.06.2018	18	56	21	52	18 h 50 min
25.06.2018	19	61	21	64	18 h 49 min
26.06.2018	21	65	20	71	18 h 49 min
27.06.2018	22	71	21	78	18 h 48 min
28.06.2018	21	78	19	73	18 h 46 min
29.06.2018	19	82	21	62	18 h 46 min
30.06.2018	20	58	16	65	18 h 44 min
01.07.2018	16	68	15	74	18 h 42 min
02.07.2018	20	73	16	83	18 h 41 min
03.07.2018	23	71	18	74	18 h 38 min
04.07.2018	26	66	20	71	18 h 36 min
05.07.2018	25	65	20	76	18 h 34 min
06.07.2018	20	71	19	87	18 h 32 min
07.07.2018	21	85	19	83	18 h 29 min
08.07.2018	23	80	20	77	18 h 26 min
09.07.2018	21	79	19	83	18 h 23 min
10.07.2018	21	79	20	80	18 h 20 min
11.07.2018	27	71	23	76	18 h 17 min
12.07.2018	31	60	24	66	18 h 14 min
13.07.2018	30	63	24	67	18 h 10 min
14.07.2018	29	58	24	65	18 h 8 min
15.07.2018	33	47	25	61	18 h 4 min

16.07.2018	34	45	27	67	18 h 0 min
17.07.2018	35	53	28	68	17 h 56 min
18.07.2018	34	53	28	59	17 h 52 min
19.07.2018	34	52	27	66	17 h 49 min
20.07.2018	32	63	25	77	17 h 45 min
21.07.2018	25	82	24	83	17 h 41 min
22.07.2018	28	74	24	79	17 h 37 min
23.07.2018	26	82	23	81	17 h 32 min
24.07.2018	30	63	24	71	17 h 27 min
25.07.2018	30	58	25	68	17 h 23 min
26.07.2018	28	64	23	74	17 h 19 min
27.07.2018	33	55	27	69	17 h 15 min
28.07.2018	32	63	25	70	17 h 9 min
29.07.2018	30	63	25	71	17 h 5 min
30.07.2018	32	76	26	80	17 h 1 min
31.07.2018	37	91	25	99	16 h 55 min
01.08.2018	29	95	25	100	16 h 51 min
02.08.2018	31	100	26	100	16 h 46 min
03.08.2018	31	100	25	100	16 h 41 min
04.08.2018	30	95	24	100	16 h 36 min
05.08.2018	26	100	23	100	16 h 30 min
06.08.2018	27	100	22	100	16 h 26 min
07.08.2018	27	97	21	95	16 h 21 min
08.08.2018	28	100	22	96	16 h 16 min
09.08.2018	30	100	25	100	16 h 11 min
10.08.2018	32	100	25	100	16 h 5 min
11.08.2018	27	100	22	100	16 h 0 min
12.08.2018	21	81	19	96	15 h 55 min
13.08.2018	24	76	19	88	15 h 50 min
14.08.2018	23	74	19	83	15 h 45 min
15.08.2018	21	77	18	94	15 h 39 min
16.08.2018	24	81	19	84	15 h 34 min
17.08.2018	26	75	20	75	15 h 29 min
18.08.2018	27	71	22	71	15 h 24 min
19.08.2018	23	62	19	58	15 h 18 min
20.08.2018	24	73	19	100	15 h 13 min
21.08.2018	20	86	17	99	15 h 8 min
22.08.2018	23	75	17	87	15 h 2 min
23.08.2018	22	88	19	100	14 h 57 min
24.08.2018	26	84	20	99	14 h 51 min
25.08.2018	24	99	19	93	14 h 46 min
26.08.2018	19	81	17	96	14 h 41 min
27.08.2018	23	99	18	76	14 h 35 min
28.08.2018	23	94	18	85	14 h 31 min
29.08.2018	18	88	17	99	14 h 26 min
30.08.2018	20	84	17	89	14 h 20 min
31.08.2018	20	78	17	96	14 h 15 min
01.09.2018	20	62	19	30	14 h 10 min
02.09.2018	22	80	19	98	14 h 4 min
03.09.2018	23	99	20	95	13 h 59 min

04.09.2018	22	96	19	90	13 h 54 min
05.09.2018	21	91	18	65	13 h 48 min
06.09.2018	22	49	19	66	13 h 43 min
07.09.2018	25	98	20	99	13 h 38 min
08.09.2018	23	99	19	99	13 h 32 min
09.09.2018	21	100	17	99	13 h 27 min
10.09.2018	19	94	15	89	13 h 22 min
11.09.2018	18	92	16	60	13 h 16 min
12.09.2018	20	98	16	93	13 h 11 min
13.09.2018	18	99	15	96	13 h 6 min
14.09.2018	18	99	15	94	13 h 0 min
15.09.2018	20	99	15	59	12 h 55 min
16.09.2018	18	98	13	99	12 h 50 min
17.09.2018	17	89	14	66	12 h 44 min
18.09.2018	21	88	16	43	12 h 39 min
19.09.2018	24	83	17	71	12 h 33 min
20.09.2018	23	92	17	70	12 h 27 min
21.09.2018	22	98	18	27	12 h 22 min
22.09.2018	18	99	14	56	12 h 17 min
23.09.2018	17	96	12	75	12 h 11 min
24.09.2018	15	97	11	79	12 h 6 min
25.09.2018	15	77	10	89	12 h 1 min
26.09.2018	11	65	10	74	11 h 55 min
27.09.2018	15	90	10	83	11 h 50 min
28.09.2018	12	92	8	97	11 h 44 min
29.09.2018	13	74	8	89	11 h 39 min
30.09.2018	10	93	10	95	11 h 34 min
01.10.2018	14	99	10	96	11 h 28 min
02.10.2018	18	77	16	77	11 h 23 min
03.10.2018	18	73	16	78	11 h 17 min
04.10.2018	17	54	15	55	11 h 12 min
05.10.2018	16	62	17	70	11 h 7 min
06.10.2018	19	68	18	70	11 h 1 min
07.10.2018	17	72	15	65	10 h 56 min
08.10.2018	19	55	17	62	10 h 50 min
09.10.2018	19	64	19	64	10 h 45 min
